# Microbial Community Structures and Dynamics in the O_3_/BAC Drinking Water Treatment Process

**DOI:** 10.3390/ijerph110606281

**Published:** 2014-06-16

**Authors:** Jian Tian, Jun Lu, Yu Zhang, Jian-Cheng Li, Li-Chen Sun, Zhang-Li Hu

**Affiliations:** 1 Shenzhen Key Laboratory of Marine Bioresource and Eco-environmental Science, Shenzhen Engineering Laboratory of Marine Algal Biotechnology, College of Life Science, Shenzhen University, Shenzhen 518060, China; E-Mails: terrence.tian@hotmail.com (T.J.); jun.lu@aut.ac.nz (J.L.); biozy@szu.edu.cn (Z.Y.); lijc1007@163.com (J.-C.L.); sunlch@mail2.sysu.edu.cn (L.-C.S.); 2 Faculty of Health & Environmental Sciences, and Institute of Biomedical Technology, Auckland University of Technology, Auckland 1142, New Zealand

**Keywords:** drinking water, advanced treatment, bacteria, microbial community structures

## Abstract

Effectiveness of drinking water treatment, in particular pathogen control during the water treatment process, is always a major public health concern. In this investigation, the application of PCR-DGGE technology to the analysis of microbial community structures and dynamics in the drinking water treatment process revealed several dominant microbial populations including: *α-Proteobacteria*, *β-Proteobacteria*, *γ-Proteobacteria, Bacteroidetes*, *Actinobacteria Firmicutes* and *Cyanobacteria*. *α-Proteobacteria* and *β-Proteobacteria* were the dominant bacteria during the whole process. *Bacteroidetes* and *Firmicutes* were the dominant bacteria before and after treatment, respectively. *Firmicutes* showed season-dependent changes in population dynamics. Importantly, *γ-Proteobacteria*, which is a class of medically important bacteria, was well controlled by the O_3_/biological activated carbon (BAC) treatment, resulting in improved effluent water bio-safety.

## 1. Introduction

During the past decade, the advances in biological detection technologies and the accumulation of epidemiological data have deepened our understanding of the risks generated by aquatic pathogenic microorganisms. Microbial safety of drinking water has attracted more and more attention and has become one of the most important goals for water quality management. Advanced ozone/biological activated carbon (O_3_/BAC) treatment, which is a popular method to remove organic compounds in drinking water, can increase the biological stability of the water pipe network. However, a considerable number of bacteria-based microbes can survive, grow, and accumulate on the surface of the carbon particles. These microbes will be subsequently eluted from BAC and might constitute a microbial risk for drinking water safety. In water treatment systems, factors affecting microbial growth and reproduction include turbidity, assimilable organic carbon (AOC) concentration, dissolved oxygen (DO) concentration, water temperature, pH, retention time, chlorine concentration, ammonia concentration and other physical and chemical indicators [1]. These factors change in different processing stages of the water treatment, resulting in dynamic changes in water bacterial community structures. 

Previous report indicated that only 0.1% to 1% of microbes in natural environment can be cultured by conventional methods [2], with the vast majority being in a viable but non-culturable state (VBNC). Under VBNC and the following revival status, pathogenicity still exist in some water-borne pathogens, such as *Vibrio cholerae* [3], *Shigella dysenteriae* type1 [4], and enterotoxigenic *E. coli* (*E.* *coli* H10407) [5]. However, traditional micro-ecological analysis, which mainly analyzes the culturable microbes, can hardly detect bacterium under these status and might not be accurate enough to reflect the real community structure and dynamic of environment samples. Therefore, it is necessary to determine specific microbial targets in addition to the usual water quality markers (total microbial count/L, coliform counts/L, *etc*.) during water treatment. Since the first application of denaturing gradient gel process electrophoresis (DGGE) technology in microbial ecology studies [6], it has been developed into one of the major molecular methods in this field and has been used to examine samples from a variety of sources, such as oceans [7], soils [8], sewage (waste) waters [9], reservoirs [10], rivers and lakes [11], food [12], drinking waters [13], animal guts [14], plant rhizospheres and leaves [15], and activated sludges [16]. DGGE has shown obvious advantages over convention methods in studying dominant microbial community structure, genetic diversity and population dynamics. This is especially the case when the target of the study is whole-microbial communities.

By comparing the bacterial communities from samples of ground water and bottled mineral water, Dewettinck *et al*., confirmed the existence of VBNC bacteria in all of the samples tested [17]. Importantly, the community diversity is a lot higher in ground water as compared with bottled mineral water samples, indicating the necessity of monitoring water treatment efficiency using DGGE. However, there are very limited studies on micro-ecological changes in the processes of advanced drinking water treatment, resulting in the lack of systematic data for microbial safety assessment. This study aims to investigate the microbial community structures and dynamics during the process of large-scale O3/BAC drinking water treatment. The results should provide important information on whether microbial leakage is a problem in those established large-scale O_3_/BAC water treatment plants running in the downstream area of the Pearl River, southern China. As such, it may also provide experimental basis for the prediction and control of water microbiological risks. 

## 2. Experimental Section

### 2.1. Sample Collection

Water samples were collected once each quarter from Shenzhen Meilin (SZML) drinking water treatment plant located in Shenzhen City, southern China. They were labelled in accordance with the water process procedure as follows: (a) source water, (b) pre-ozone-treated effluent, (c) flocculation and sedimentation tank effluent, (d) sand-filter-pool effluent, (e) main ozone treatment tank effluent, (f) active-charcoal-pool effluent, and (g) factory effluent. Twenty liters of water sample from each treatment step were collected and immediately taken back to the Institute of Eco-Environmental Science at Shenzhen University. The subsequent DNA extraction and PCR were carried out using an identical reaction system and conditions.

### 2.2. Total DNA Extraction

Bacteria in water samples were collected by vacuum filtration using a PALL polyethersulfone filtration membrane with pore size of 0.22 μm. The membranes were stored at −20 °C until use. Genomic DNA of the bacteria was extracted using the OMEGA EZNA Water DNA Kit (Omega Bio-Tek, Norcross, GA, USA) according to the manufacturer’s protocol.

### 2.3. PCR-DGGE Analysis

To obtain the bacterial community structures in different samples, V3 regions of 16S ribosomal DNA (rDNA) were amplified using a set of 16S rDNA domain specific primers (primer F, 5GCCCG-CCGCGCGCGGCGGGCGGGGCGGGGGCACGGGGGGCCTACGGGAGGCAGCAG and primer R, ATTACCGCGGCTGCTGG). A touchdown PCR reaction programme, with 0.5 °C lower annealing temperature for each cycle, was used in this study. The targeted amplification segment was about 200 bp in length. DGGE was performed using a DCode Universal Mutation Detection System (Bio-Rad, Hercules, CA, USA). The DGGE gel concentration was determined to be 10% with 35–70% denaturant gradient range. An equal volume (40 μL) of PCR product from each sample was loaded onto gel and electrophoresis was run at constant 75 V voltage for around 14 h. The gels were imaged after 20 min GelRed (Biotium, Hayward, CA, USA) staining. The images were analyzed with the Quantity One software (Bio-Rad). 

### 2.4. 16S rDNA Sequencing and Community Structure Analysis

The dominant bands in the DGGE gel were labeled, cut, placed in a 1.5 mL tube with 100 μL of deionized water. The mixture was incubated at 65 °C for 1 h. An aliquot of 2 μL of the extracted solution was used as a template and amplified using non-GC-clamp primers of the 16S rDNA V3 region. The amplified products were sequenced by the Shenzhen Huada Genomics Institute. The sequences were blasted against the Genebank database and the most similar matches were obtained. Relative abundances were assigned to specific sequences base on the appropriate band intensity relative to the total intensity of the profile. The DGGE profiles were normalized based on sample volume and the community structure diagram for each sample was drawn according to the collected trace values and the percentage of content.

## 3. Results and Discussion

### 3.1. Composition of Bacteria Community at Different Stage of Water Treatment

**Figure 1:**
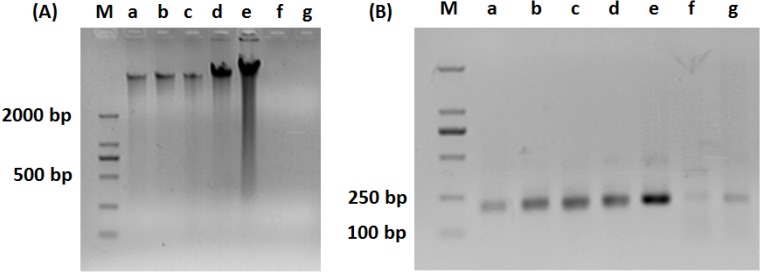
Agrose gel electrophoresis of (**A**) total DNA extracted from water sample and (**B**) PCR products of 16S rDNA. M: maker; **a**: source water; **b**: pre-ozone-treated effluent; **c**: flocculation and sedimentation tank effluent; **d**: sand-filter-pool effluent; **e**: main ozone treatment tank effluent; **f**: active-charcoal-pool effluent; **g**: factory effluent.

As shown in Figure 1A, DNA was efficiently extracted from the water samples of the first five stages of water treatment, including (a) source water, (b) pre-ozone-treated effluent, (c) flocculation and sedimentation tank effluent, (d) sand-filter-pool effluent, and (e) main ozone treatment tank effluent. In contrast, no apparent band could be detected in lane (f) and (g), indicating DNA concentrations are low in the last two stages of water treatment. As shown in Figure 1B, specific bands at size of 200 bp were detectable from all of the samples after PCR amplification. However, the densities of lane (f) and (g) were much weaker than the rest of the samples.

**Figure 2:**
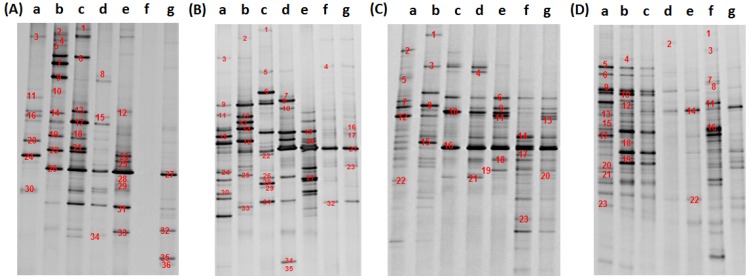
DGGE patterns of 16S rDNA fragments amplified from water sample DNA with primer V3. (**A**) Feburary, (**B**) May, (**C**) August, (**D**) November. **a**: source water; **b**: pre-ozone-treated effluent; **c**: flocculation and sedimentation tank effluent; **d**: sand-filter-pool effluent; **e**: main ozone treatment tank effluent; **f**: active-charcoal-pool effluent; **g**: factory effluent.

DGGE fingerprint analysis detected 35, 40, 36 and 41 dominant bands from the water samples collected in February, May, August, and November, respectively (Figure 2). Results indicate that the diversity of bacterial community in each sample was relatively high (except for sample f in February). Some bands existed consistently in multiple samples, indicating that the treatment processes have limited effect on removing these species. For example, the 21st band in the May sample and the 16th band in the August sample appeared across samples from different stages of treatment. Similar bands included the 26th and 31st in February, 6th and 10th in August, and 9th, 14th and 22nd in November. Some of the bands disappear and reappear in different treatment stages, such as the 3rd and 33rd bands in February and the 26th band in May, indicating the bacterial removal efficiency of different processes may vary depending on the characteristics of the treatment method itself. The high-density bands which represent the dominant microbial populations showed the differences in microbial structure of the various water samples. The differences in localization of dominant bands in the adjacent lanes suggested that there were various degrees of changes in the microbial community structure after different water treatment process procedures.

By pairwise comparison of the bands between lanes in the DGGE map, the similarity of the microbial community in the different processes could be determined. The difference between the lane c and d was the lowest, with a similarity ratio of 55.1%, 70% and 35.5% for February, August and November, respectively, indicating minimal impact of the sand filtration process on the bacterial community structures. The difference between lane f and g was small, with a similarity ratio of 87.4% and 67.0% in May and August, respectively. There was a relatively big difference between lane f and g in February and November, suggesting that clear pool storage and activated charcoal treatment had little effect on bacterial survival in water during summer and autumn, but had some influence during spring and winter, presumably related to water temperature. Eichler *et al.* also found considerable stability of the bacteria community structure in pool water with little change for a long period of time [18]. However, they did not discuss its relationship with temperature.

Overall, the diversity of the bacterial community did not show a continuous decrease with water treatment procedures. The diversity of the bacterial community was rich in source water under various seasonal conditions, but not necessarily the most abundant compared with water samples from other treatment steps. The bacterial community structures and composition show big differences among water samples processed with different technologies. For example, the dominant bacteria community in samples b, e and f changed significantly compared with that in the effluent of the previous treatment step, suggesting that there is a significant impact of ozone and activated charcoal treatments on the water bacteria community structures and turnover. This maybe directly caused by the strong sterilization effect of ozone. Compared with pre-processed water samples, the bacterial community diversity in sample f decreased in February and May, but increased in August and November, possibly due to the leakage of micro-organisms off the activated charcoal filtration pool.

In total, 117 bands were sequenced (Table S1) for GeneBank database comparison. Among these, 76 were matched with sequence similarity greater than 98%. Forty nine of them are culturable bacteria, while the remaining 68 were non-culturable types. Only 14 out of 117 could be assigned to specific species (sequence similarity greater than 97%), accounting for 12% of total samples. Water flora in the whole process was divided into seven categories, namely: (1) *α-Proteobacteria*, including species such as *Methylobacterium*, *Sphingomonas,* *Novosphingobium, Porphyrobacter* and *Erythrobacter*; (2) *β-Proteobacteria*, including species such as *Methylophilus, Hydrogenophaga, Acidovorax, Oxalobacteraceae* and some taxa of *Comamonadaceae*; (3) *γ-Proteobacteria*, including genera or species such as *Pseudomonas* *aeruginosa* strain, *Pseudomonas* *anguilliseptica* etc.; (4) *Bacteroidetes*, including *Flavobacterium* and some other unclassified non-culturable groups; (5) *Firmicutes*, mainly *Bacillus*, groups of *Lactococcus*; (6) *Actinobacteria* and (7) *Cyanobacteria*. We also detected a small amount of *Deltaproteobacteria, Acidobacteria* and *Chloroflexi*. Most of these floras are well-defined water and soil borne bacteria, many of which are commonly present in drinking water pipe network and water supply systems [19]. Many of the others have similar sequences to bacteria from freshwater lakes, rivers and drinking water treatment plants. 

Among the flora existing across the drinking water treatment process, some of them can effectively degrade macromolecular organic pollutants, such as aromatic hydrocarbons and pesticides, including *Sphingomonas,* *Novosphingobium,* *Pseudomonas sp,* *Hydrogenophag.* Liu *et al*. isolated a new type of the bacterium *Novosphingobium taihuense sp*., from sediments of Taihu Lake, China, which is able to degrade some chemicals, such as phenol, aniline, nitrobenzene, and phenanthrene [20]. Some species are associated with lake eutrophication, such as *Cyanobacterium,* *Duganella* *sp.* and *Lacibacter* *cauensis* *strain*. *Duganella* *sp*. (AM989091.1) was isolated from lake water and defined during the study of heterotrophic bacteria diversity in algal bloom [21]. *Lacibacter* *cauensis* *strain* (EU521690.1) was isolated from the bottom sediment of eutrophic lakes [22].

### 3.2. Analysis of Water Bacteria Community Dynamics at Various Process Stages

**Figure 3:**
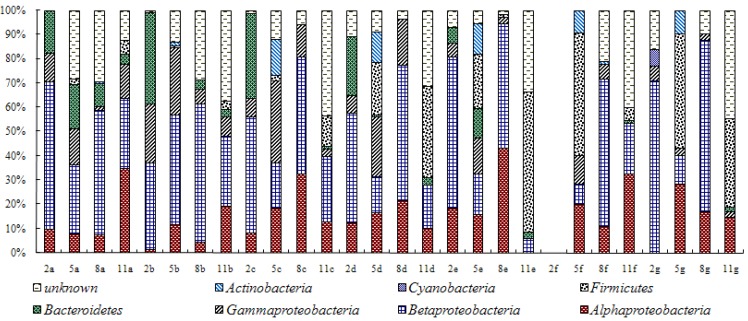
Composition of the major phylogenetic groups found in water sample during different stages of advanced drinking water treatment. 2: February; 5: May, 8: August; 11: November. **a**: source water; **b**: pre-ozone-treated effluent; **c**: flocculation and sedimentation tank effluent; **d**: sand-filter-pool effluent; **e**: main ozone treatment tank effluent; **f**: active-charcoal-pool effluent; **g**: factory effluent.

Different treatment process had different effects on the bacterial community dynamics. Due to the quality of the source water, bacterial community structures varied in samples from effluent of each treatment stage in different months. In general (Figure 3), during the different treatment stages, the contents of *α-Proteobacteria* and *β-Proteobacteria* were relatively stable and were not influenced by the treatment and showed no seasonal change. Contents of *Bacteroides* and *γ-Proteobacteria* sharply decreased after the ozone and activated charcoal filtration treatments, possibly due to the fact these two types of bacteria were more sensitive to ozone’s strong oxidation. The bacterial content in factory effluent was greatly reduced compared to the samples from early treatment stages. The *γ-Proteobacteria* include some important groups of medical importance, such as *Enterobacteraceae, Vibrionaceae* and *Pseudomonadaceae*. A number of important pathogens belong to this class, such as *Salmonella* (causes enteritis and typhoid fever), *Yersinia* (causes the plague), *Vibrio* (causes cholera), *Pseudomonas aeruginosa*, *etc*. The sequence analysis found the opportunistic pathogen of *Pseudomonas* *aeruginosa* strain a few times. Although the *γ-Proteobacteria* can be detected in factory effluent, the inactivation of these bacteria by the ozone has guaranteed the safety of drinking water to certain extent. *Firmicutes* content changed seasonally, as it could be detected in summer and winter but not in spring and autumn, and remained low during the first four stages. Unlike *γ-Proteobacteria*, *Firmicutes* appeared after ozone treatment and gradually became the dominant bacteria, possibly due to its higher sensitivity to TOC and increased dissolved oxygen content. *Actinomycetes* could only be detected in the samples collected in May and their content was stable. As they are a group of soil borne bacteria, it is suggested that their specific appearance may be associated with heavy rainfall in summer. Content of *Cyanobacteria* was the lowest and could only be detected in factory effluent in February.

Due to the limitations of the DGGE technique, it can only detect DNA fragments from 200 to 1,000 bp. Using such a short sequence for data mining and comparison, a lot of bacteria could only be identified to the family or genus level. Therefore, the content of pathogens may be either overestimated [23] or underestimated [24]. Moreover, the possibility of multiple sequences within a band may also be considered. However, DGGE has been proven to generate data that are in good agreement with those from the classic culture-dependent methods and the most recent next generation sequencing methods [25,26]. As indicated in our study, PCR-DGGE is a robust exploratory approach to target specific microbial contaminants during the O_3_/BAC water treatment process. In the future, an RNA-based quantitative real-time PCR technique will be used to give more accurate bacterial community diversity results during drinking water treatment.

## 4. Conclusions

The current study has revealed seven dominant bacteria groups in O_3_/BAC treated drinking water, including *α-Proteobacteria*, *β-Proteobacteria*, *γ-Proteobacteria*, *Bacteroidetes*, *Actinobacteria*, *Firmicutes* and *Cyanobacteria*. Most of them are commonly defined freshwater and soil borne bacteria and are commonly found in drinking water pipe networks and water supply systems.

*α-Proteobacteria* and *β-Proteobacteria* were the dominant bacteria throughout the water treatment process*.* *Bacteroides* and *Firmicutes* were stage-specific and were the dominant bacteria in the early and later treatment stages. The dynamics of *Firmicutes* showed seasonal changes. The presence of important pathogenic microbes, namely *γ-Proteobacteria*, was well controlled by activated charcoal/ozone treatment. In summary, our results clearly indicate the usefulness of PCR-DGGE methodology in monitoring bacterial community dynamics during drinking water treatment. The bacterial profiling data generated in this study may also aid in the development of quantitative PCR primers in future studies.

## Supplementary Files

Supplementary File 1 Supplementary Information (PDF, 138 KB)

## References

[1] Niquette P., Servais P., Savoir R. (2001). Bacterial dynamics in the drinking water distribution system of Brussels. Water Res..

[2] Ward D.M., Bateson M.M., Weller R., Ruff-Roberts A.L. (1992). Ribosomal RNA analysis of microorganisms as they occur in nature. Adv. Microb. Ecol..

[3] Colwell1 R.R., Brayton P.R., Grimes D.J., Roszak D.B., Huq S.A., Palmer L.M. (1985). Viable but non-culturable *Vibrio cholerae* in the environment: Implications for release of genetically engineered microorganisms. Nat. Biotechnol..

[4] Rahman I., Shahamat M., Kirchman P.A., Russek-Cohen E., Colwell R.R. (1994). Methionine uptake and cytopathogenicity of viable but nonculturable *Shigella dysenteriae type 1*. Appl. Environ. Microbiol..

[5] Pommepuy M., Butin M., Derrien A., Gourmelon M., Colwell R.R., Cormier M. (1996). Retention of enteropathogenicity by viable but nonculturable *Escherichia coli* exposed to seawater and sunlight. Appl. Environ. Microbiol..

[6] Muyzer G., de Waal E.C., Uitterlinden A.G. (1993). Profiling of complex microbial populations by denaturing gradient gel electrophoresis analysis of polymerase chain reaction amplified genes encoding for 16S rRNA. Appl. Environ. Microbiol..

[7] Li Z.Y., He L.M., Miao X.L. (2007). Cultivable Bacterial Community from South China Sea Sponge as Revealed by DGGE Fingerprinting and 16S rDNA Phylogenetic Analysis. Curr. Microbiol..

[8] Kandeler E., Tscherko D., Bruce K.D., Stemmer M., Hobbs P.J., Bardgett R.D., Amelung W. (2000). Structure and function of the soil microbial community in microhabitats of a heavy metal polluted soil. Biol. Fertil. Soils.

[9] Paniagua-Michel J., Franco-Rivera A., Cantera J.J.L., Stein L.Y. (2005). Activity of Nitrifying Biofilms Constructed on Low-Density Polyester Enhances Bioremediation of a Coastal Wastewater Effluent. World J. Microb. Biot..

[10] Wang J., Ma T., Zhao L., Lv J., Li G., Zhang H., Zhao B., Liang F., Liu R. (2008). Monitoring exogenous and indigenous bacteria by PCR-DGGE technology during the process of microbial enhanced oil recovery. J. Ind. Microbiol. Biotechnol..

[11] Pearce D.A. (2003). Bacterioplankton Community Structure in a Maritime Antarctic Oligotrophic Lake during a Period of Holomixis, as Determined by Denaturing Gradient Gel Electrophoresis (DGGE) and Fluorescence in Situ Hybridization (FISH). Microb. Ecol..

[12] Wang H.Y., Zhang X.J., Zhao L.P. (2008). Analysis and comparison of the bacterial community in fermented grains during the fermentation for two different styles of Chinese liquor. J. Ind. Microbiol. Biotechnol..

[13] Wu Q., Zhao X.H., Zhao S.Y. (2006). Application of PCR-DGGE in Research of Bacterial Diversity in Drinking Water. Biomed. Environ. Sci..

[14] Molnár O., Wuczkowski M., Prillinger H. (2008). Yeast biodiversity in the guts of several pests on maize;comparison of three methods: classical isolation, cloning and DGGE. Mycol. Progress.

[15] Garbeva P., Overbeek L.S., Vuurde J.W., Elsas J.D. (2001). Analysis of endophytic bacterial communities of potato by plating and denaturing gradient gel electrophoresis (DGGE) of 16S rDNA based PCR fragments. Microb. Ecol..

[16] Wang X.H., Zhang K., Ren N.Q., Li N., Ren L.J. (2009). Monitoring microbial community structure and succession of an A/O SBR during start-up period using PCR-DGGE. J. Environ. Sci..

[17] Dewettinck T., Hulsbosch W., Van Hege K., Top E.M., Verstraete W. (2001). Molecular fingerprinting of bacterial populations in groundwater and bottled mineral water. Appl. Microbiol. Biotechnol..

[18] Eichler S., Christen R., Höltje C., Westphal P., Bötel J., Brettar I., Mehling A., Höfle M.G. (2006). Composition and Dynamics of Bacterial Communities of a Drinking Water Supply System as Assessed by RNA-and DNA-Based 16S rRNA Gene Fingerprinting. Appl. Environ. Microbiol..

[19] Schmeisser C., Stöckigt C., Raasch C., Wingender J., Timmis K.N., Wenderoth D.F., Flemming H.C., Liesegang H., Schmitz R.A., Jaeger K.E., Streit W.R. (2003). Metagenome Survey of Biofilms in Drinking-Water Networks. Appl. Environ. Microbiol..

[20] Liu Z.P., Wang B.J., Liu Y.H., Liu S.J. (2005). Novosphingobium taihuense sp nov., a novel aromatic-compound-degrading bacterium isolated from Taihu Lake, China. Int. J. Syst. Evol. Microbiol..

[21] Berg K.A., Lyra C., Sivonen K., Paulin L., Suomalainen S., Tuomi P., Rapala J. (2009). High diversity of cultivable heterotrophic bacteria in association with cyanobacterial water blooms. ISME. J..

[22] Qu J.H., Yuan H.L., Yang J.S., Li H.F., Chen N. (2009). *Lacibacter cauensis gen.nov., sp.nov.,* a novel member of the phylum Bacteroidetes isolated from sediment of a eutrophic lake. Int. J. Syst. Evol. Microbiol..

[23] Muyzer G., Smalla K. (1998). Application of denaturing gradient gel electrophoresis (DGGE) and temperature gradient gel electrophoresis (TGGE) in microbial ecology. A. Van. Leeuw..

[24] Yang C.H., Crowley D.E. (2000). Rhizosphere microbial community structure in relation to root location and plant iron nutritional status. Appl. Environ. Microbiol..

[25] Vaz-Moreira I., Egas C., Nunes O.C., Manaia C.M. (2013). Bacterial diversity from the source to the tap: A comparative study based on 16S rRNA gene-DGGE and culture-dependent methods. FEMS. Microbiol. Ecol..

[26] Delgado S., Rachid C.T., Fernández E., Rychlik T., Alegría A., Peixoto R.S., Mayo B. (2013). Diversity of thermophilic bacteria in raw, pasteurized and selectively-cultured milk, as assessed by culturing, PCR-DGGE and pyrosequencing. Food Microbiol..

