# Supplementary material for: Microbial Community Structures and Dynamics in the O3/BAC Drinking Water Treatment Process
Source: Int J Environ Res Public Health. 2014 Jun 16;11(6):6281–90. doi: 10.3390/ijerph110606281 (PMC4078579; doi:10.3390/ijerph110606281)
Supplement: Supplementary File 1 — Supplementary Information (PDF, 138 KB) [file ijerph-11-06281-s001.pdf]

Supplementary Information

Microbial Community Structures and Dynamics in the O_3/BAC Drinking Water Treatment Process

---

Table S1. Sequence similarities to closest relatives and phylogenetic affiliations of DNA recovered from DGGE gel.

Bands	Accession No.	Closest 16S rRNA Gene Sequence (Accession No.)	Similarity	Taxonomic Group	Isolation Source
ADW2-1	HQ730491	*Flavobacterium sp*. CJ4(EU099872.1)	97%	*Bacteroidetes*	Freshwater lake
ADW2-2	HQ730492	Uncultured bacterium clone LM83(AM909976.1)	100%	*β-Proteobacteria*	Chinese rice field
ADW2-3	HQ730493	*Oxalobacteraceae* bacterium AKB-2008-JO53(AM989125.1)	96%	*β-Proteobacteria*	Lake water
ADW2-4	HQ730494	Uncultured gamma proteobacterium clone VERDEA24(FJ902591.1)	98%	*γ-Proteobacteria*	Biofilm
ADW2-5	HQ730495	Uncultured gamma proteobacterium clone LPROCKB32(FJ902308.1)	96%	*γ-Proteobacteria*	Biofilm
ADW2-6	HQ730496	Uncultured *Flavobacterium sp*.(FN179352.1)	97%	*Bacteroidetes*	Lake water
ADW2-7	HQ730497	Gamma proteobacterium A62-2(AY701876.1)	97%	*γ-Proteobacteria*	Freshwater
ADW2-8	HQ730498	*Sediminibacterium sp*. TEGAF015(AB470450.1)	99%	*Bacteroidetes*	Freshwater
ADW2-9	HQ730499	Gamma proteobacterium A62-2(AY701896.1)	97%	*γ-Proteobacteria*	Freshwater
ADW2-10	HQ730500	Uncultured *Pseudomonas sp*.(AM711878.1)	98%	*γ-Proteobacteria*	Water and feces
ADW2-11	HQ730501	*Collimonas sp*. NCCB 100025(AY281149.1)	98%	*β-Proteobacteria*	
ADW2-12	HQ730502	*Pseudomonas sp*. RF-122(GQ205106.1)	100%	*γ-Proteobacteria*	Canal
ADW2-13	HQ730503	*Pseudomonas anguilliseptica* strain S1(NR_029319.1)	97%	*γ-Proteobacteria*	
ADW2-14	HQ730504	*Pseudomonas aeruginosa* strain SML(GQ915373.1)	98%	*γ-Proteobacteria*	Soil
ADW2-15	HQ730505	Uncultured *Bacteroidetes* bacterium clone MA00186F08(FJ532511.1)	98%	*Bacteroidetes*	Soil
ADW2-16	HQ730506	*Methylophilus methylotrophus* strain CS3(GQ175365.1)	97%	*β-Proteobacteria*	Tobacco-planted soil
ADW2-17	HQ730507	*Flavobacterium johnsoniae* strain 451-1(FN298315.1)	98%	*Bacteroidetes*	Casts of earthworms
ADW2-18	HQ730508	*Flavobacterium sp*.HI-M4(DQ205296.1)	98%	*Bacteroidetes*	Cavern
ADW2-19	HQ730509	*Pseudomonas sp*. TSSAS2-17(GQ284545.1)	98%	*γ-Proteobacteria*	Mangrove sediment
ADW2-20	HQ730510	Uncultured bacterium clone 16S-KM-B-48(AB238057.1)	96%	*β-Proteobacteria*	Drinking water
ADW2-21	HQ730511	*Flavobacterium sp*. HMD1070(GU291856.1)	98%	*Bacteroidetes*	
ADW2-22	HQ730512	Beta proteobacterium JPPB B32(GU368378.1)	97%	*β-Proteobacteria*	Drinking water

Table S1. *Cont.*

Bands	Accession No.	Closest 16S rRNA Gene Sequence (Accession No.)	Similarity	Taxonomic Group	Isolation Source
ADW2-23	HQ730513	Uncultured bacterium clone nbw672b10c1(GQ112460.1)	98%	*β-Proteobacteria*	Skin, popliteal fossa
ADW2-24	HQ730514	Uncultured bacterium clone 16S-KM-B-27(AB238036.1)	98%	*β-Proteobacteria*	Drinking water
ADW2-25	HQ730515	*Limnobacter thioxidans* strain TSWCSN35(GQ284439.1)	98%	*β-Proteobacteria*	Natural spring sediment
ADW2-26	HQ730516	Uncultured bacterium clone nbu289a11c1(GQ020946.1)	98%	*β-Proteobacteria*	Skin, volar forearm
ADW2-27	HQ730517	Uncultured bacterium clone nbw701a06c1(GQ098665.1)	100%	*β-Proteobacteria*	Skin, volar forearm
ADW2-28	HQ730518	Uncultured bacterium clone ncd368d12c1(HM320473.1)	99%	*β-Proteobacteria*	Skin
ADW2-29	HQ730519	Uncultured bacterium clone ncd368d12c1(HM320473.1)	100%	*β-Proteobacteria*	Skin
ADW2-30	HQ730520	*Novosphingobium sp.* S23301(D84598.2)	97%	*α-Proteobacteria*	
ADW2-31	HQ730521	Uncultured *Comamonadaceae* bacterium clone Gap-1-54(EU642073.1)	100%	*β-Proteobacteria*	Milwaukee harbor
ADW2-32	HQ730522	*Methylobacterium sp.* JPPB A3(GU368371.1)	100%	*α-Proteobacteria*	Drinking water
ADW2-33	HQ730523	Beta proteobacterium HIBAF006(AB452985.1)	99%	*β-Proteobacteria*	Freshwater lake
ADW2-34	HQ730524	*Flavobacterium sp.* HMD2009(HM135522.1)	99%	*Bacteroidetes*	Lake water
ADW2-35	HQ730525	*Methylobacterium sp.* 5b.1.1(FJ157968.1)	100%	*α-Proteobacteria*	Plant surfaces
ADW2-36	HQ730526	Uncultured *cyanobacterium* clone as1-58(GU257586.1)	97%	*Cyanobacteria*	Activated sludge
ADW5-1	HQ730527	Uncultured bacterium clone 1F06(AY537705.1)	100%	*γ-Proteobacteria*	Danio rerio digestive tract
ADW5-2	HQ730528	Uncultured bacterium clone DGGE band R2-27(AB194359.1)	92%	*β-Proteobacteria*	Lake water
ADW5-3	HQ730529	Uncultured gamma proteobacterium clone VERDEA24(FJ902591.1)	99%	*γ-Proteobacteria*	Biofilm
ADW5-4	HQ730530	Uncultured *Lactococcus sp.* clone OTUN3(EU826672.1)	97%	*Firmicutes*	Beef
ADW5-5	HQ730531	Uncultured *Methylophilaceae* bacterium clone GASP-MB1S3_G01(EF664718.1)	98%	*β-Proteobacteria*	Soil
ADW5-6	HQ730532	*Pseudomonas anguilliseptica* strain S1(NR_029319.1)	98%	*γ-Proteobacteria*	
ADW5-7	HQ730533	*Pseudomonas sp.* AKB-2008-HE49(AM989280.1)	98%	*γ-Proteobacteria*	Baltic Sea
ADW5-8	HQ730534	*Pseudomonas sp.* AKB-2008-HE54(AM989281.1)	99%	*γ-Proteobacteria*	Baltic Sea
ADW5-9	HQ730535	*Pseudomonas aeruginosa* strain SML(GQ915373.1)	98%	*γ-Proteobacteria*	Soil
ADW5-10	HQ730536	*Methylophilus methylotrophus* strain CS3(GQ175365.1)	99%	*β-Proteobacteria*	Soil
ADW5-11	HQ730537	Uncultured *Sphingobacteriales* bacterium clone GASP-KC2S1_G12(EU299585.1)	99%	*α-Proteobacteria*	Grassland soil
ADW5-12	HQ730538	Uncultured *Pseudomonas sp.* clone F3Baug.28(GQ417851.1)	100%	*γ-Proteobacteria*	Degreasing systems
ADW5-13	HQ730539	*Duganella sp.* AKB-2008-TA4(AM989091.1)	99%	*β-Proteobacteria*	Lake water
ADW5-14	HQ730540	Uncultured bacterium clone 16S-KM-B-48(AB238057.1)	98%	*β-Proteobacteria*	Drinking water

Table S1. *Cont.*

Bands	Accession No.	Closest 16S rRNA Gene Sequence (Accession No.)	Similarity	Taxonomic Group	Isolation Source
ADW5-15	HQ730541	Sphingomonas sp. PXM 16S(AY232825.1)	98%	*α-Proteobacteria*	Soil
ADW5-16	HQ730542	Uncultured *Micrococcineae* bacterium clone Jab PL2W2G8(HM486303.1)	96%	*Actinobacteria*	Wetlands
ADW5-17	HQ730543	Uncultured *Bacteriodetes* bacterium clone MA194T1-3r5_H07(GQ469438.1)	98%	*Bacteroidetes*	Soil
ADW5-18	HQ730544	*Flavobacterium sp*. ARSA-15(GU295972.1)	100%	*Bacteroidetes*	
ADW5-19	HQ730545	Uncultured bacterium clone 16S-KM-B-37(AB238046.1)	100%	*β-Proteobacteria*	Drinking water
ADW5-20	HQ730546	Uncultured Comamonadaceae bacterium clone Gap-1-54(EU642073.1)	97%	*β-Proteobacteria*	Milwaukee harbor
ADW5-21	HQ730547	Uncultured Bacillus sp.(EF032879.1)	98%	*Firmicutes*	Rhizosphere soil
ADW5-22	HQ730548	Uncultured gamma proteobacterium clone saw_2_46(FJ854715.1)	98%	*γ-Proteobacteria*	Water
ADW5-23	HQ730549	*Methylobacterium sp*. MAFF 211642(AB518685.1)	97%	*α-Proteobacteria*	Soil
ADW5-24	HQ730550	*Novosphingobium sp*. BD-2(AB377503.1)	100%	*α-Proteobacteria*	
ADW5-25	HQ730551	*Mitsuaria chitosanitabida* strain JC6(FJ609679.1)	100%	*β-Proteobacteria*	Sewage
ADW5-26	HQ730552	*Sphingomonas sp*. Asd M4-14(FM955867.1)	100%	*α-Proteobacteria*	Sandy sediment
ADW5-27	HQ730553	Uncultured gamma proteobacterium clone LPROCKB92(FJ902331.1)	97%	*γ-Proteobacteria*	Limestone sinkholes
ADW5-28	HQ730554	Uncultured bacterium clone MRM1027(FN428773.1)	99%	*β-Proteobacteria*	River water
ADW5-29	HQ730555	Uncultured bacterium clone CL48STW5(FJ205055.1)	97%	*β-Proteobacteria*	Well water
ADW5-30	HQ730556	*Lacibacter cauensis* strain NJ-8(EU521690.1)	99%	*Bacteroidetes*	Lake sediment
ADW5-31	HQ730557	*Sphingopyxis sp*. KYH-1(AB235163.1)	99%	*α-Proteobacteria*	
ADW5-32	HQ730558	*Methylobacterium sp*. 5b.1.1(FJ157968.1)	98%	*α-Proteobacteria*	Plant surfaces
ADW5-33	HQ730559	*Methylobacterium radiotolerans* strain 1019(GU294334.1)	100%	*α-Proteobacteria*	Leaf
ADW5-34	HQ730560	Uncultured bacterium clone MBR-8_HF_BF53(FM201201.1)	97%	*γ-Proteobacteria*	Membrane bioreactors
ADW5-35	HQ730561	Uncultured *Bacteroidetes* bacterium clone DE1A2(FJ916830.1)	96%	*Bacteroidetes*	Lake epilimnion
ADW8-1	HQ730562	Uncultured bacterium clone UTFS-R12-90-67(GQ871560.1)	95%	*Bacteroidetes*	Sluge
ADW8-2	HQ730563	Uncultured proteobacterium clone CFBC2H11(GU127197.1)	98%	*β-Proteobacteria*	Reservoir
ADW8-3	HQ730564	*Pseudomonas aeruginosa* strain NRRL B-14935(DQ459316.1)	99%	*γ-Proteobacteria*	
ADW8-4	HQ730565	Uncultured *Methylophilus sp*. clone 2-53(GQ464401.1)	98%	*β-Proteobacteria*	Frozen chicken
ADW8-5	HQ730566	Uncultured *Sphingobacteriales* bacterium clone GASP-KC2S1_G12(EU299585.1)	96%	*α-Proteobacteria*	Grassland
ADW8-6	HQ730567	Uncultured bacterium clone K29C2-4(AB504638.1)	97%	*β-Proteobacteria*	DHS reactor

Table S1. *Cont.*

Bands	Accession No.	Closest 16S rRNA Gene Sequence (Accession No.)	Similarity	Taxonomic Group	Isolation Source
ADW8-7	HQ730568	Uncultured bacterium isolate DGGE gel band 12(GQ356024.1)	100%	*Bacteroidetes*	Activated sludge
ADW8-8	HQ730569	Uncultured bacterium clone 16S-KM-B-48(AB238057.1)	98%	*β-Proteobacteria*	Drinking water
ADW8-9	HQ730570	*Erythrobacter sp*. YCSD40(FJ984821.1)	99%	*α-Proteobacteria*	Farm Sediment
ADW8-10	HQ730571	*Porphyrobacter sp*. MA112(FJ377384.1)	98%	*α-Proteobacteria*	
ADW8-11	HQ730572	*Limnobacter thioxidans* strain TSWCSN35(GQ284439.1)	98%	*β-Proteobacteria*	Spring sediment
ADW8-12	HQ730573	*Acidovorax sp*. GPTSA100-27(DQ854967.1)	97%	*β-Proteobacteria*	
ADW8-13	HQ730574	*Bacillus sp*. CC-CCM15-5(EU689094.1)	98%	*Firmicutes*	Rhizosphere
ADW8-14	HQ730575	Uncultured *Comamonadaceae* bacterium clone P8s-213(GQ329490.1)	91%	*β-Proteobacteria*	Soil
ADW8-15	HQ730576	Uncultured *Methyloversatilis sp*. isolate DGGE gel band B8(FJ750463.1)	96%	*β-Proteobacteria*	Sluge
ADW8-16	HQ730577	Uncultured *Hydrogenophaga sp*. isolate DGGE band 19(EU026424.1)	96%	*β-Proteobacteria*	Lake water
ADW8-17	HQ730578	Uncultured bacterium isolate DGGE gel band BUJIT10(GQ245707.1)	97%	*γ-Proteobacteria*	Landfill leachate
ADW8-18	HQ730579	Uncultured *Comamonadaceae* bacterium clone Gap-1-54(EU642073.1)	95%	*β-Proteobacteria*	Milwaukee harbor
ADW8-19	HQ730580	Uncultured alpha proteobacterium isolate DGGE band WETLE-14R(FM992018.1)	97%	*α-Proteobacteria*	Wetland
ADW8-20	HQ730581	Uncultured *Pseudomonas sp*. clone CAR3(FJ868262.1)	99%	*γ-Proteobacteria*	Sluge
ADW8-21	HQ730582	Uncultured *Hydrogenophaga* sp. clone P1s-266(GQ287425.1)	95%	*β-Proteobacteria*	Soil
ADW8-22	HQ730583	Uncultured actinobacterium clone 2K91(GU074275.1)	99%	*Actinobacteria*	Groundwater
ADW8-23	HQ730584	*Comamonadaceae* bacterium CNRF14(GU300568.1)	97%	*β-Proteobacteria*	Soil
ADW11-1	HQ730585	Uncultured *Sphingopyxis sp*.clone BF.A160(FM174857.1)	99%	*α-Proteobacteria*	Rivulet
ADW11-2	HQ730586	Uncultured *Lactococcus sp*. clone OTUN3(EU826672.1)	98%	*Firmicutes*	Refrigerated beef
ADW11-3	HQ730587	*Flavobacterium sp*. A14 strain A14-B19(AF406663.1)	98%	*Bacteroidetes*	
ADW11-4	HQ730588	Uncultured bacterium clone 5C231434(EU803802.1)	98%	*ε-Proteobacteria*	Lake Gatun
ADW11-5	HQ730589	*Pseudomonas aeruginosa* strain 1S280(EF427781.1)	100%	*γ-Proteobacteria*	
ADW11-6	HQ730590	Uncultured alpha proteobacterium clone zxh-8-36(GU323679.1)	98%	*α-Proteobacteria*	Freshwater lake
ADW11-7	HQ730591	Uncultured *Bdellovibrio sp*.clone CL2.C81(FM175407.1)	96%	*δ-Proteobacteria*	Rivulet
ADW11-8	HQ730592	Uncultured bacterium clone PM1_a02d05(GQ493804.1)	95%	*Firmicutes*	Mice cecal
ADW11-9	HQ730593	Uncultured bacterium clone 16S-KM-B-48(AB238057.1)	88%	*β-Proteobacteria*	Drinking water
ADW11-10	HQ730594	Uncultured bacterium clone DP7.1.101(FJ612244.1)	96%	*α-Proteobacteria*	Lake water
ADW11-11	HQ730595	Uncultured *Sphingopyxis sp*.clone CL3.D3(FM175750.1)	100%	*α-Proteobacteria*	Rivulet

Table S1. *Cont.*

Bands	Accession No.	Closest 16S rRNA Gene Sequence (Accession No.)	Similarity	Taxonomic Group	Isolation Source
ADW11-12	HQ730596	Gamma proteobacterium GPTSA100-26(DQ914846.1)	98%	*γ-Proteobacteria*	
ADW11-13	HQ730597	Uncultured bacterium clone 16S-KM-B-37(AB238046.1)	98%	*β-Proteobacteria*	Drinking water
ADW11-14	HQ730598	Uncultured *Bacillus sp.*clone K39(FN434378.1)	98%	*Firmicutes*	Deteriorated stone
ADW11-15	HQ730599	Uncultured bacterium clone 5C230913(EU803354.1)	95%	*Acidobacteria*	Lake Gatun
ADW11-16	HQ730600	Uncultured bacterium clone Z123(GQ388799.1)	94%	*β-Proteobacteria*	Drinking water
ADW11-17	HQ730601	*Bosea sp*. TSA6w(AB542375.1)	98%	*α-Proteobacteria*	Rice paddy soil
ADW11-18	HQ730602	*Sphingomonadales* bacterium LN13-TH(FJ572029.1)	100%	*α-Proteobacteria*	Lake water
ADW11-19	HQ730603	Uncultured freshwater bacterium clone 965004D12.x1(DQ065377.1)	99%	*α-Proteobacteria*	Freshwater
ADW11-20	HQ730604	Uncultured bacterium DGGE band1-2(AY071872.1)	95%	*ε-Proteobacteria*	Changjiang River
ADW11-21	HQ730605	Uncultured alpha proteobacterium clone ARTE4_259(GU230191.1)	98%	*α-Proteobacteria*	Coastal water
ADW11-22	HQ730606	*Exiguobacterium aurantiacum* strain NH88-23(FJ447534.1)	98%	*Firmicutes*	Ocean
ADW11-23	HQ730607	Uncultured *Chloroflexi* bacterium clone CYN-1-16(EU240732.1)	96%	*Chloroflexi*	Freshwater

© 2014 by the authors; licensee MDPI, Basel, Switzerland. This article is an open access article distributed under the terms and conditions of the Creative Commons Attribution license (http://creativecommons.org/licenses/by/3.0/).
